# Portable Micro-Doppler Radar with Quadrature Radar Architecture for Non-Contact Human Breath Detection

**DOI:** 10.3390/s21175807

**Published:** 2021-08-28

**Authors:** Catur Apriono, Fathul Muin, Filbert H. Juwono

**Affiliations:** 1 Department of Electrical Engineering, Faculty of Engineering, Universitas Indonesia, Depok 16424, Indonesia; fathul.muin@ui.ac.id; 2 Department of Electrical and Computer Engineering, Curtin University Malaysia, Miri 98009, Malaysia; filbert@ieee.org

**Keywords:** micro-Doppler radar, breathing rate detection, Vivaldi antenna

## Abstract

Recently, rapid advances in radio detection and ranging (radar) technology applications have been implemented in various fields. In particular, micro-Doppler radar has been widely developed to perform certain tasks, such as detection of buried victims in natural disaster, drone system detection, and classification of humans and animals. Further, micro-Doppler radar can also be implemented in medical applications for remote monitoring and examination. This paper proposes a human respiration rate detection system using micro-Doppler radar with quadrature architecture in the industrial, scientific, and medical (ISM) frequency of 5.8 GHz. We use a mathematical model of human breathing to further explore any insights into signal processes in the radar. The experimental system is designed using the USRP B200 mini-module as the main component of the radar and the Vivaldi antennas working at 5.8 GHz. The radar system is integrated directly with the GNU Radio Companion software as the processing part. Using a frequency of 5.8 GHz and USRP output power of 0.33 mW, our proposed method was able to detect the respiration rate at a distance of 2 m or less with acceptable error. In addition, the radar system could differentiate different frequency rates for different targets, demonstrating that it is highly sensitive. We also emphasize that the designed radar system can be used as a portable device which offers flexibility to be used anytime and anywhere.

## 1. Introduction

Radio detection and ranging (radar) technology is a system for detecting objects using reflected radio waves’ radiation. The radar transmitter sends a radio wave that is reflected when hitting an object. The reflecting wave is then captured by the receiver to be further processed. The obtained information from the reflected wave can be used to determine the location of the object and its movement. Therefore, radar is commonly used for both military (e.g., air surveillance) and civilian (e.g., weather surveillance) purposes [1,2].

Thanks to advancements in electronics, mechanics, wireless-component, and signal-processing and -computing technologies [3,4,5,6,7,8,9,10], the radar system has been investigated for a wide range of applications, such as navigation, aerospace, space exploration, and medical. In the navigation sector, radar plays a vital role in tracking aircraft movements in order to prevent congestion and unexpected accidents [11,12]. A cognitive radar system was designed for an autonomous robot in [13]. Further, radar can also be used in automotive applications for self-parking, blind-spot monitoring, etc. [14]. The concept of an aperture radar for space exploration with low mass and power consumption was introduced in [15].

For the past few decades, the medical field has utilized radar systems, such as for tumor detection, breath monitoring, heartbeat monitoring, and the detection of buried victims in natural disasters [16,17,18,19,20]. Many research works in the healthcare sector focused on detecting the function of vital human organs, such as the lungs and heart, as they are essential health indicators [7,21,22,23,24,25,26]. In fact, radar technology is very useful for medical instruments, as it can provide nonphysical contact between patient and examination unit. As a consequence, it provides a more practical and safe method for carrying out the patient’s examination, especially in some particular conditions where the patient cannot be touched (e.g., burn wounds) [27]. The authors in [28] suggested a radar device designed to detect the pulse and breathing of human beings from a distance of 30 m. Results demonstrated improved accuracy compared to that of other approaches, such as direct-contact sensors and optical cameras.

The Doppler radar technique is commonly used for the detection of the human respiratory rate (breathing) [29]. The Doppler radar transmitter emits a continuous-wave (CW) signal in a specific direction to free space, while the receiver detects a reflected wave from the human-chest plane due to breathing. The phase of the reflected waves differs from the original phase because of the object’s (chest) movement. In general, the different positions of the target at a net-zero velocity represent phase-modulated signals corresponding to the direction of the target. In the case of breathing, the movement of the chest leads to the modulation of the emitted signal, thus allowing for the reflected wave to convey information about the movement of the chest. In other words, a continuous-wave radar detects a phase-modulated signal due to the movement of the chest, which oscillates as a function of time. After the radar receiver captures the reflected wave, the respiratory rate is estimated by demodulating the signal in the frequency domain.

The use of Doppler radar for the detection of the breathing rate has contributed to a variety of research projects since 1975. Not only for direct static uses, the technique was also extended to a larger range of applications, such as the discovery of victims of falling building debris and human identification behind walls [30,31,32,33]. A technique using a string to measure chest expansion was introduced in [34]. The proposed method could be used to monitor respiratory function and certain other health parameters. However, the system was controlled by directly touching the human body. In addition, it was difficult to obtain the correct extracted pieces of information during the configuration process.

A particular phenomenon called a zero point is a common problem observed in radar science. When the signals on the local oscillator and receiver are either inphase or 180 degrees out of phase, detection accuracy can be decreased. A quadrature radar can be used to reduce the null-point effect [35].

While the most recent research has shown remarkable results, the use of a conventional radar system has several issues, including physical size and clutter issues [36]. Some research works established methods to improve the quality of the measured signal. A common way is to eliminate signal clutter picked up by the radar. This signal clutter comes from objects around the radar, reflecting the signal emitted by the radar. The reflection of the signal caused by objects around the radar can reduce the desired signal quality. However, vital-sign detection measurements that are carried out have a short distance. This is because the input power generated by these components is very limited. Conventional radar systems are not suitable for home and portable applications because they are large and use expensive components.

Radar research can now be carried out using silicon radar chip technology with advanced semiconductor technologies [37,38,39,40,41]. Almost all physical-layer elements, such as the local oscillator, voltage controller oscillator (VCO), analog-to-digital converter (ADC), amplifier, filter, and antennas, are already accessible in a single chip. The chip radar device is ideal for home-surveillance applications due to its compactness, and ease of installation and calculation. This silicon chip also provides excellent measurement results; the distance that can be measured using this system is also up to 3 m. However, the fabrication of this system is very complex because it involves many components.

As we have the same source for transmission and detection, the phase noise of observed signals can be correlated with the local oscillator. High correlation would minimize the quality of the detected signal. Fortunately, software-defined radio (SDR), such as the USRP module released by National Instrument, can be used to minimize the phase noise on the silicon chips. SDR is a software-based operation in which a user can operate and change parameters just on the software integrated with GNU Radio, LabVIEW, or MATLAB. Therefore, it is easy to adjust variables such as frequency, gain, and sample rate. The authors in [42] used the SDR module to detect breathing activities with various conditions by using omnidirectional radiation antennas. The use of omnidirectional antennas can result in reduced gain, which is unlikely to be resolved by the transmit power. As a result, the system was able to operate over a short distance of about 40 cm. Furthermore, the system could detect many clutter signals from other signals.

In this paper, we designed a novel quadrature radar system to detect human breathing rate in the industrial, scientific, and medical (ISM) frequency of 5.8 GHz using directional antennas. We chose the 5.8 GHz ISM frequency band since the amplitude of chest oscillation due to breathing is proportional to the quarter wavelength of the frequency, and the frequency allows for using small-dimension antennas [43]. In particular, we used Vivaldi antennas and a USRP B200mini module (from Ettus Research Laboratory) as the SDR [44]. Our proposed system can detect human breathing rates up to a distance of 2 m using low USRP output power and directional Vivaldi antennas to improve the ability of the system to minimize clutter signals caused by foreign objects around the radar. We summarize the contributions of our paper as follows:

- The use of directional Vivaldi antennas can minimize clutter around the radar, thereby improving the quality of breathing detection.
- Human breathing detection up to a distance of 2 m with low USRP power, i.e., 0.33 mW.
- Phase-noise reduction, as the USRP B200mini module can help to reduce phase noise generated by leakage currents in electronic components.

The rest of this paper is organized as follows. Section 2 discusses the quadrature radar system. The system design along with the components are presented in Section 3. The experimental results and discussion are given in Section 4. Lastly, Section 5 concludes this paper.

## 2. Quadrature Doppler Radar

As mentioned above, micro-Doppler radar is useful to detect weak movements and vibrations in many applications, such as detecting breathing rates through time-varying movements of the chest. The micro-Doppler radar system transmits a CW signal through the air. When the signal hits the chest plane, it is reflected off and induced to change its phase according to the chest movement. Doppler theory states that an object with a time-varying position and net zero velocity reflects the signal with its phase modulated according to the position movement.

The transmitted signal, which is sometimes called the local-oscillator (LO) signal, with unity amplitude is given by [37]

(1) T(t)=cos(2πft+φ(t)),

where *f* denotes the oscillator center frequency, *t* is the elapsed time, and φ(t) is the phase noise generated by the oscillator. The single-band CW signal propagates towards the human body with a distance of $d_{0}$ from the transmitter.

When the CW signal hits the chest plane, some portion is reflected towards the radar, and the rest is absorbed by the human body. Let x(t) be the time-varying chest movement (displacement) due to breathing. The ideal reflected signal captured by the receiver is expressed by [37]

(2) $$R\left(t\right)=\cos\left[2\pi ft-\frac{4\pi d_{0}}{\lambda }-\frac{4\pi x(t)}{\lambda }+\varphi \left(t-\frac{2d_{0}}{c}\right)\right],$$

where λ=c/f is the wavelength of the oscillator signal, and *c* is the velocity of the electromagnetic wave. (2) shows that the received signal does not change in frequency. Further, it has a time-varying phase term as a function of x(t), and time delay as a function of the distance between radar and chest. R(t) and LO signals are then mixed and low-filtered to produce baseband signal B(t). Further, the quadrature radar can be achieved by decomposing the baseband signal into inphase (I) and quadrature (Q) components as follows.

(3) $$B_{I}\left(t\right)=\cos\left(\theta +\frac{\pi }{4}+\frac{4\pi x(t)}{\lambda }+\Delta \varphi \left(t\right)\right),$$

(4) $$B_{Q}\left(t\right)=\cos\left(\theta -\frac{\pi }{4}+\frac{4\pi x(t)}{\lambda }+\Delta \varphi \left(t\right)\right),$$

where

(5) $$\theta =\frac{4\pi d_{0}}{\lambda }+\theta_{0},$$

$\theta_{0}$ is a constant affected by the shift at the reflection surface and distance between mixer and antenna. In (3) and (4), Δφ(t) is the residual phase noise given by

(6) $$\Delta \varphi \left(t\right)=\varphi \left(t\right)-\varphi \left(t-\frac{2d_{0}}{c}\right).$$

The LO generates phase noise, as indicated by the equations above. Oscillator signals can go out of phase due to phase noise. The LO and reflected signals must be in phase (0 degree) or in quadrature (90 degrees) to minimize phase noise and improve detection accuracy.

According to the null-point principle, either the I or Q component can be used to determine the breathing rate. As chest displacement corresponds to breathing rate, (5) is generally proportional to the breathing rate.

## 3. System Design

**Figure 1:**
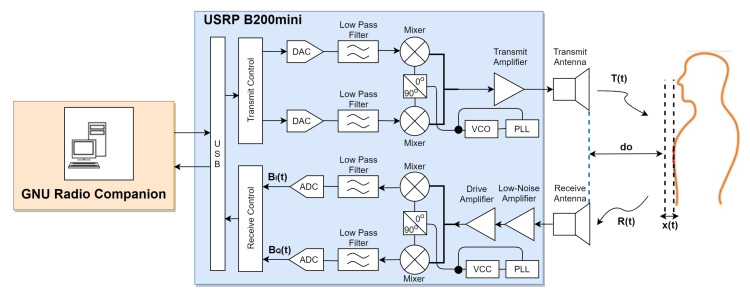
Block diagram of quadrature radar architecture.

The quadrature radar architecture is shown in Figure 1. This experimental system design consisted of the following components: a signal generator, transmit and receive antennas, an analog-to-digital converter (ADC), and a signal processor. These components are represented by USRP B200mini Device, Vivaldi antennas, a breath vibrator, and GNU Radio Companion.

### 3.1. USRP B200mini Device

USRP is a radio-frequency device designed for a number of SDR-related applications. The USRP module consists of SDR components and has an architecture that performs signal-processing functions on other host devices via USB2.0. In this architecture, the system is similar to the quadrature radar architecture. Some other components, such as ADC, filter, mixer, VCO, and amplifier are available in the module. Therefore, it can be easily adjusted according to the requirement without changing the hardware components.

### 3.2. Vivaldi Antenna

**Figure 2:**
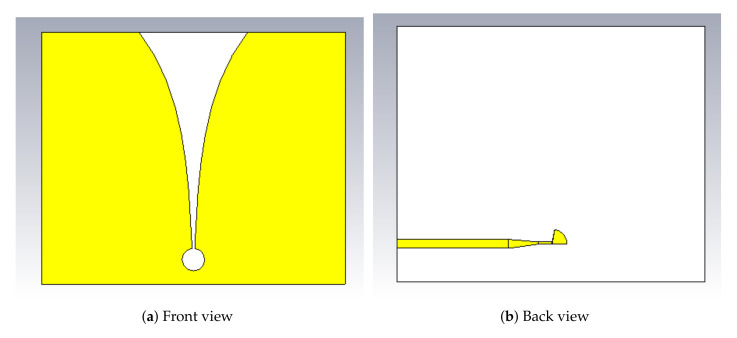
Front and back views of Vivaldi antenna.

**Figure 3:**
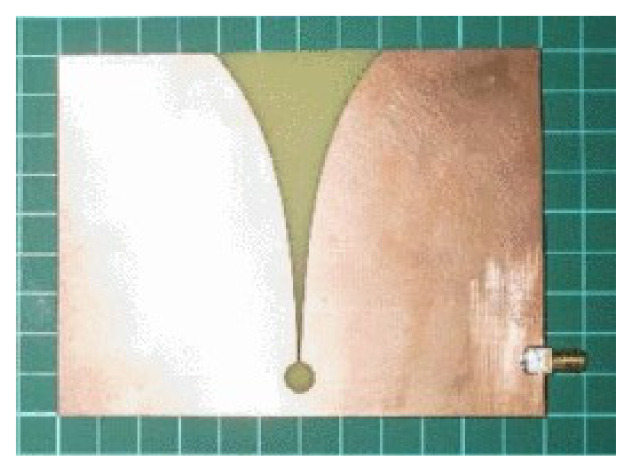
Vivaldi antenna used in the proposed radar system [45].

In the experiment, Vivaldi antennas were used as transmit and receive antennas. We used the Vivaldi antenna proposed in [45]. The front and back views of the design are illustrated in Figure 2. The fabricated antenna is shown in Figure 3. As shown in Table 1, the dimensions of the antenna supported the development of a compact radar system. The Vivaldi antenna was designed using FR-4 substrate with a dielectric constant of $\epsilon_{r}=4.3$, working frequency of 5.8 GHz, and a return loss of −24.62 dB.

**Figure 4:**
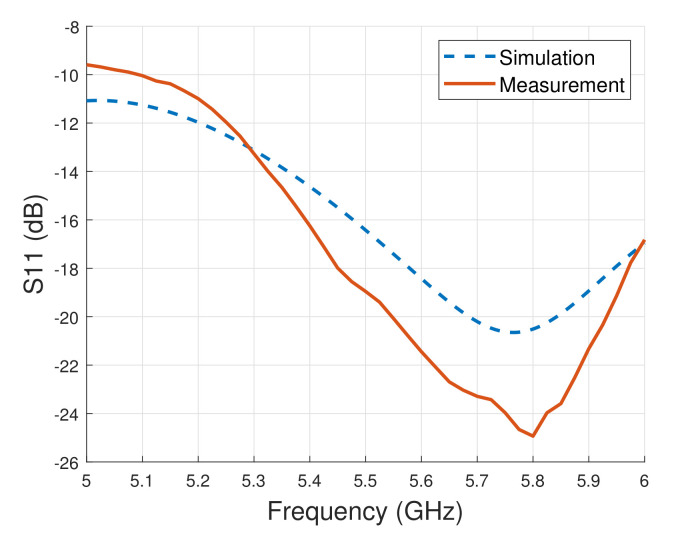
Return loss, simulation vs. measurement.

Figure 4 shows simulation and measurement results of the return loss. The antenna works in wide frequencies of 5.12–6.20 GHz with a bandwidth of 1.08 GHz. The return loss was measured using an Agilent N5230C network analyzer. Figure 4 shows that the simulated return loss at the frequency of 5.8 GHz was −20.59 dB, while the measured return loss was −24.62 dB. The measurement result was thus better than the simulation results.

**Figure 5:**
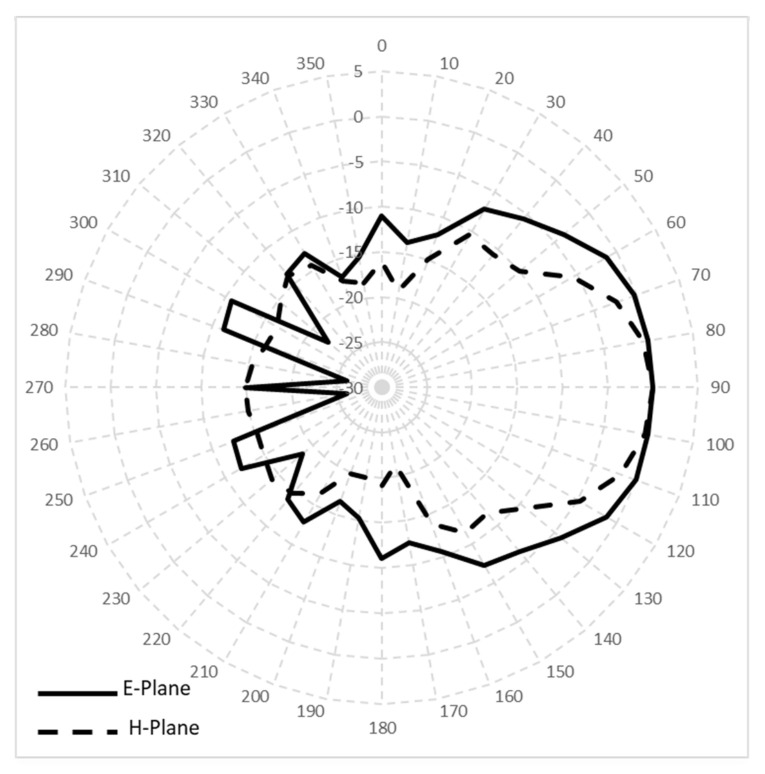
Radiation patterns of Vivaldi antenna.

Figure 5 shows the directional radiation pattern at 5.8 GHz with E-plane half-power beamwidth of 73.2 degrees and H-plane half-power beamwidth of 46.6 degrees. The measured gain was 4 dBi. The antenna performance indicated that it is suitable for the proposed application.

### 3.3. Breath Vibrator

**Figure 6:**
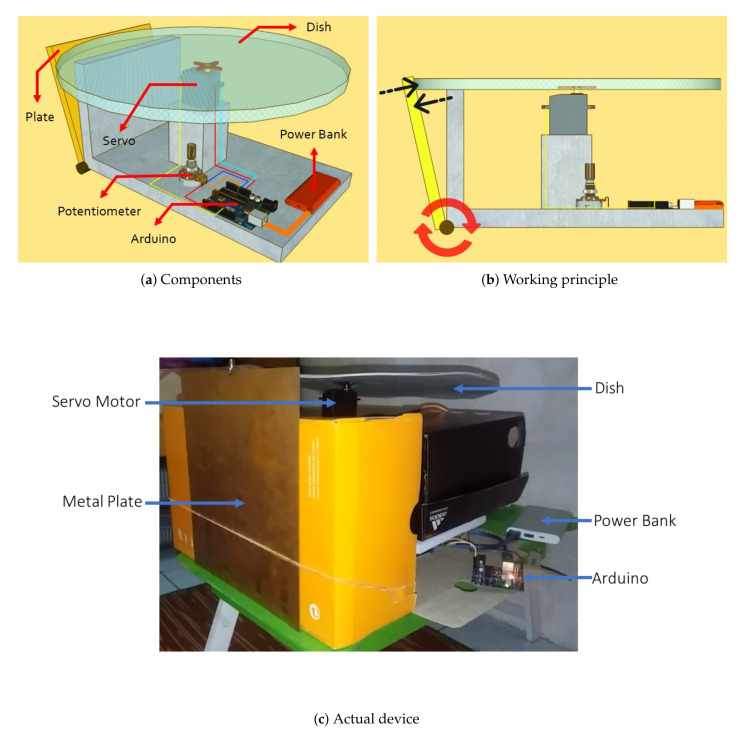
Breath-vibrator design.

A breath vibrator is a device that generates vibration, and creates interference with electromagnetic waves. It was designed to produce wave visualizations similar to human breathing. The device was used to test the proposed radar system with predetermined parameters. Figure 6a shows a schematic diagram of the breath vibrator and the components used in the device. In particular, the components used for the device include a plate, dish, servomotor, potentiometer, Arduino, and power bank. The breath vibrator activates the servomotor, which rotates the dish and allows for the plate to oscillate following the shape of the dish, as shown in Figure 6b. The actual device is shown in Figure 6c.

The motion of the plate creates a wave disruption similar to the visualization of human breathing. The breath vibrator was examined. The human breathing rate is visualized by the amount of revolutions per minute of the disc, which is regulated by the speed of the servomotor. The vibrator breathing system was used in the experiment as a replacement for the human target. We set the motor speed to a specific level, and ran the servo motor for a few minutes while counting the number of revolutions. Tests were carried out 20 times. Due to the servo motor’s limitations, slight variations in the number of revolutions occurred. This is covered in Section 4.1.

### 3.4. GNU Radio Companion

The GNU Radio Companion is a collection of signal-processing blocks used for radio-frequency applications that can be reviewed in real time [33]. The GNU Radio Companion can act as an independent software package or as a back end to hardware devices. This software is written in C++ and Python, so that it can be operated on Linux, Mac OSX, and Windows.

**Figure 7:**
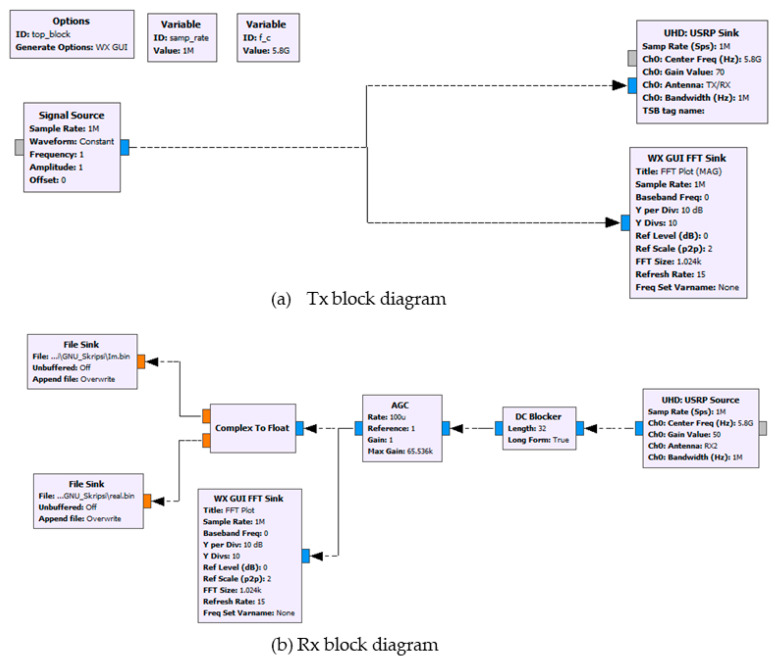
GNU block diagrams.

In the experiment, programming in GNU Radio was visually performed using block diagrams containing algorithms for processing data. The output generated from all data processing in GNU Radio was saved in a binary file. Furthermore, the binary file was processed by using MATLAB. The GNU block diagrams are depicted in Figure 7.

## 4. Results and Discussion

Two experiments were conducted in this research. The first experiment used a breath vibrator as the target object, while the second experiment used a human as the target object. The experiment using a breath vibrator as the target object was aimed to theoretically test the designed radar detection system. The system was then validated by using a human as the test object. The experiment setting and parameter values are summarized in Table 2.

The output power of the USRP significantly impacts the accuracy of the radar system. The measurement results are poorer as the output power transferred increases. This is due to the proximity of the transmitter and reception antennas, which causes signal coupling. This study was completed and verified in [46]. In addition, the usage of high power output on the USRP device is potentially dangerous. The long-term usage of high power output on the USRP can cause the device to heat up fast, which can cause damage to the USRP component. We found out that 0.33 mW output power is appropriate for evaluating the radar system after several iterations.

We manually calculated the breathing rate, which is proportional to the plate movement (in the first experiment) and chest movement (in the second experiment) for every experiment iteration as the reference value. We could not obtain a fix on the breathing rate due to the mechanics and practical limitations of the breath vibrator, and the activities of the volunteer, which affected their breathing pattern. The details are described in the following subsections.

### 4.1. Experiment 1: Using the Breath Vibrator

**Figure 8:**
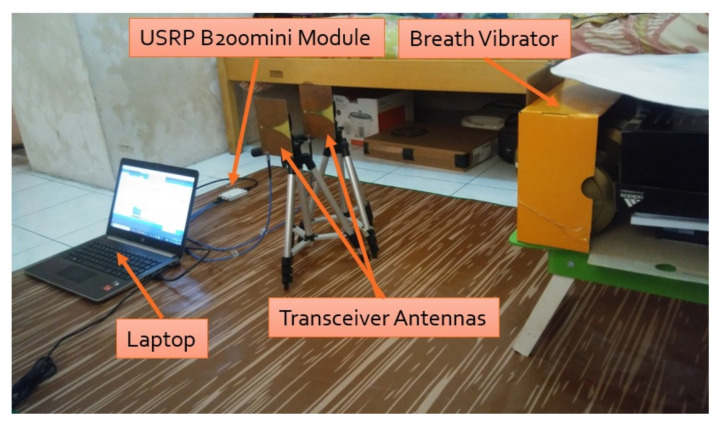
Experiment 1 setup.

Figure 8 shows the first configuration setup using the breath vibrator. In this experiment, data collection was carried out for 60 s, and observations were taken with various distances between radar and breath vibrator. The breath vibrator was measured to work at 0.35 and 0.37 Hz. There was slight difference in the reference rate due to the mechanical limitations of the servo motor. The servo motor was unable to provide a constant rpm. Two system tests were performed, with data retrieved for 1 minute under identical circumstances and rotating speed. Within 1 minute, the first test generated a metal plate vibration 21 times (0.35 Hz), whereas the second test produced it 22 times at the same rpm speed (0.37 Hz). To reduce computational complexity, the stored data were downsampled. They were then filtered to eliminate high-frequency noise. We utilized a 6th order Butterworth low-pass filter with a 2 Hz cut-off frequency. The filter coefficients were then convolved with the decimated data to produce time-domain plots, which were subsequently transformed into frequency-domain plots using Fourier transform.

**Figure 9:**
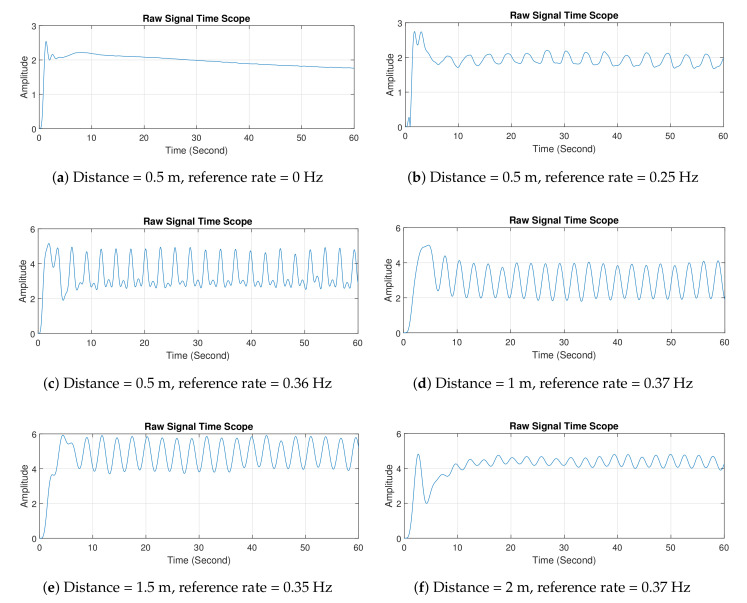
Experiment 1 results in time domain.

Figure 9 shows the measurement results in the time domain. Results show that the signal formed waves similar to sinusoidal, and noise and disturbance due to the environment. In particular, the signal was contaminated by noise and clutter around the radar, thereby leading to a distorted received signal. The waves were formed due to the metal plate’s movement of the breath vibrator. The resulting wave was weak at a distance of 2 m. In addition, at the initial time, the waves appeared to be less steady. This effect is related to the principle of path loss, namely, the greater the radar distance from the target is, the greater the resultant path loss [47]. From 0 to 5 s, graphs revealed that the produced waves were agitated. The phenomena can be explained as follows. When the radar was turned on, it captured the signal that unexpectedly enlarged, and it required time to calibrate the captured signal in order to keep the signal stable.

**Figure 10:**
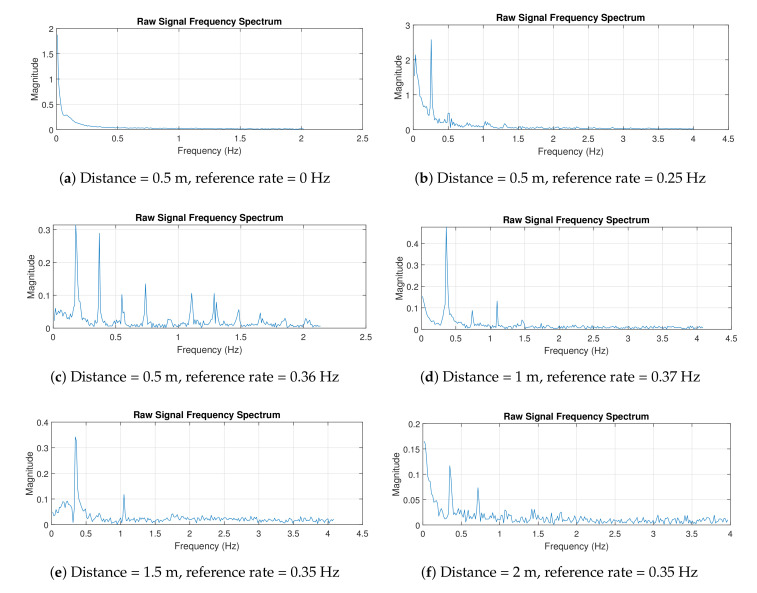
Experiment 1 results in frequency domain.

Figure 10 shows the measurement results in the frequency domain. Multiple frequencies shown in the figures were caused by some disturbances (e.g., signal clutters), so the signal was not a pure sinusoid. A pure sinusoidal signal results in a single spectral peak. Therefore, in practice, we indicate the frequency at which the highest spectral peak occurs as the breathing rate. For example, in Figure 10c, the peak occurred at a frequency of 0.3603 Hz. The designed radar was able to accurately detect the breathing rates, even when the radar was 2 m away from the target.

### 4.2. Experiment 2: Using Human Target

**Figure 11:**
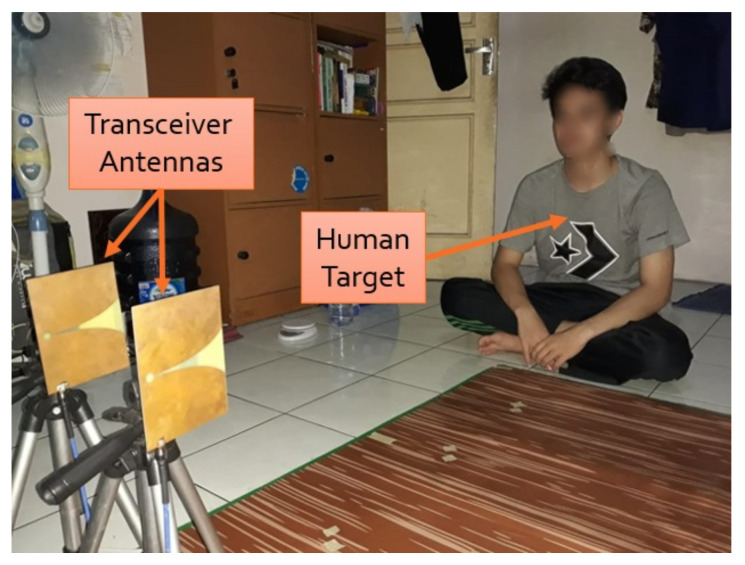
Experiment 2 setup.

After testing the radar system with a breath vibrator, we examined its detection of a human’s breathing rate. Figure 11 shows the setup of the experimental process with a volunteer. Similar to the first experiment, the distance between radar system and the human was varied. During the data-collection process, the volunteer was asked to breath normally for 60 s, and we manually counted the number of breaths to calculate the breathing rate for the reference value.

**Figure 12:**
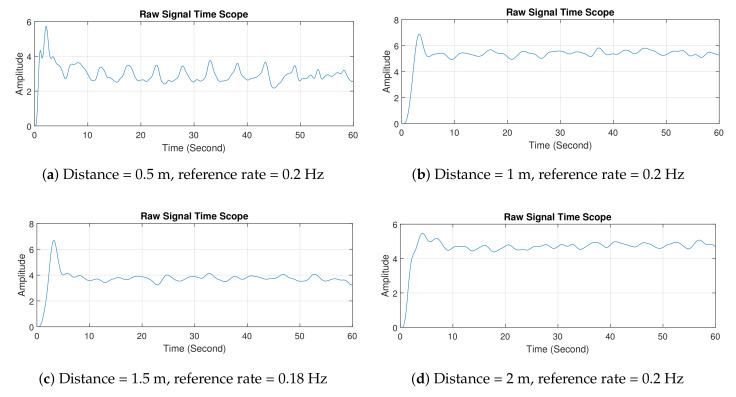
Experiment 2 results in time domain.

Figure 12 shows the experimental results of the second experiment in the time domain. Overall, the results showed that the signal formed a wave similar to a distorted sinusoidal wave. This wave signal was formed as a result of the movement of the human target’s chest. When the signal rose or went to the peak, it indicated that the human target was inhaling; when the signal dropped or went to the valley, it indicated that the human object was exhaling. Similar to the first experiment, the path loss and clutter effects proportionally increased with the distance between radar and human target.

**Figure 13:**
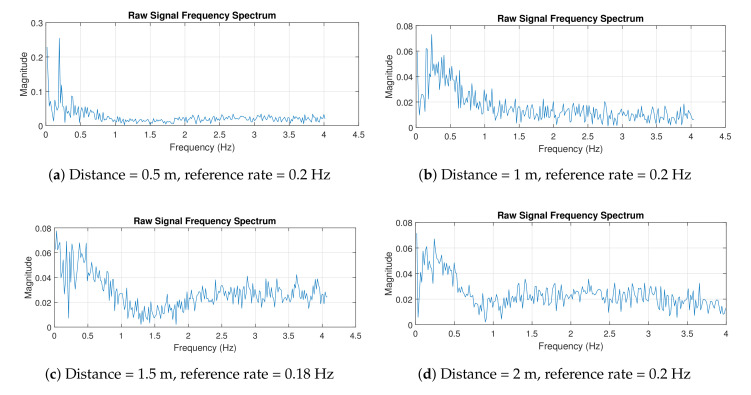
Experiment 2 results in frequency domain.

Figure 13 shows the experimental results in the frequency domain. Using the same concept as above, the breathing rate can be found by investigating the peak of the spectrum. Looking at the figures, the peak magnitude occurred within the range of 0.1–0.4 Hz. In Figure 13a, the radar system measured the breathing rate of 0.19 Hz, and the reference rate was 0.2 Hz, which produced a 5% measurement error. In contrast, a larger error of 20% was obtained when the distance between radar and human was 2 m. This error is acceptable in practice [21]. The experiment with the human target was more challenging, especially when the separation distance was large, as it is highly dependent on the human target. The target may not have a consistent breathing pattern (due to abnormal breathing problems) and may perform some other movements, e.g., head movement, which affect breathing rate and reflected signal. However, our designed radar showed some promising results that could lead to further development for biomedical applications, e.g., to detect breathing abnormalities.

## 5. Conclusions

A micro-Doppler radar with a quadrature architecture was designed to detect human breathing. The device is portable, allowing for it to be utilized in a variety of situations and settings. Experimental results revealed that the gadget could test the human breathing rate with relatively high accuracy. With low power and a range of up to 2 m, the device could detect weak vibration objects and human breathing rates. As a result, our radar technique is easily applicable for noncontact vibration-rate detection of either human breathing or vibrating objects. Furthermore, the radar system can be improved by focusing on data processing, i.e., using algorithms to filter out noise and clutter that attenuate electromagnetic signals containing information on the human respiration rate.

## Figures and Tables

**Table 1:** Dimensions of the Vivaldi antenna.

Width	89.52 mm
Length	107.68 mm
Tapered slot line	78.29 mm
Probe feed length	39.10 mm
Probe feed width	3.14 mm
Circular balun diameter	8.02 mm
Slot-line radial stub	6.67 mm

**Table 2:** Experiment setting and parameters.

Type of USRP	B200mini-i (70 MHz–6 GHz)
Carrier frequency	5.8 GHz
Antenna gain	4 dBi
Half-power beamwidth	105 deg
Bandwidth	1100 Mhz (5.1–6.2 GHz)
USRP output power	0.33 mW
Measurement distance	0.5, 1.0, 1.5, and 2.0 m
